# Machine Learning-Based Detection of Dengue from Blood Smear Images Utilizing Platelet and Lymphocyte Characteristics

**DOI:** 10.3390/diagnostics13020220

**Published:** 2023-01-06

**Authors:** Hilda Mayrose, G. Muralidhar Bairy, Niranjana Sampathila, Sushma Belurkar, Kavitha Saravu

**Affiliations:** 1 Department of Biomedical Engineering, Manipal Institute of Technology, Manipal Academy of Higher Education (MAHE), Manipal 576104, India; 2 Department of Pathology, Kasturba Medical College, Manipal Academy of Higher Education (MAHE), Manipal 576104, India; 3 Department of Infectious Diseases, Kasturba Medical College, Manipal Academy of Higher Education (MAHE), Manipal 576104, India

**Keywords:** digital pathology, dengue, machine learning, thrombocytopenia, lymphocyte, peripheral blood smear, Artificial Intelligence

## Abstract

Dengue fever, also known as break-bone fever, can be life-threatening. Caused by DENV, an RNA virus from the Flaviviridae family, dengue is currently a globally important public health problem. The clinical methods available for dengue diagnosis require skilled supervision. They are manual, time-consuming, labor-intensive, and not affordable to common people. This paper describes a method that can support clinicians during dengue diagnosis. It is proposed to automate the peripheral blood smear (PBS) examination using Artificial Intelligence (AI) to aid dengue diagnosis. Nowadays, AI, especially Machine Learning (ML), is increasingly being explored for successful analyses in the biomedical field. Digital pathology coupled with AI holds great potential in developing healthcare services. The automation system developed incorporates a blob detection method to detect platelets and thrombocytopenia from the PBS images. The results achieved are clinically acceptable. Moreover, an ML-based technique is proposed to detect dengue from the images of PBS based on the lymphocyte nucleus. Ten features are extracted, including six morphological and four Gray Level Spatial Dependance Matrix (GLSDM) features, out of the lymphocyte nucleus of normal and dengue cases. Features are then subjected to various popular supervised classifiers built using a ten-fold cross-validation policy for automated dengue detection. Among all the classifiers, the best performance was achieved by Support Vector Machine (SVM) and Decision Tree (DT), each with an accuracy of 93.62%. Furthermore, 1000 deep features extracted using pre-trained MobileNetV2 and 177 textural features extracted using Local binary pattern (LBP) from the lymphocyte nucleus are subjected to feature selection. The ReliefF selected 100 most significant features are then fed to the classifiers. The best performance was attained using an SVM classifier with 95.74% accuracy. With the obtained results, it is evident that this proposed approach can efficiently contribute as an adjuvant tool for diagnosing dengue from the digital microscopic images of PBS.

## 1. Introduction

Dengue fever is the most significant arboviral disease prevailing in many parts of the world [1,2]. According to the World Health Organization, dengue’s overall incidence has grown dangerously, causing approximately 100–400 million infections annually [3]. The occurrence of dengue has risen eight-fold over the last two decades [3]. Currently, dengue is endemic in 129 countries, posing a yearly risk to approximately 3.9 billion people [3,4]. Roughly 50% of the world’s population faces infection risk, while 70% of the total risk is in Asia [3,5]. Moreover, the combined effect of COVID 19 and dengue infections can cause destructive results in the populations at risk [3]. The virus accountable for the dengue infection is an RNA virus called DENV, consisting of four closely linked serotypes (DENV1-DENV2). These serotypes belong to the genus Flavivirus and the family Flaviviridae [6,7]. The DENV is spread through a human-to-mosquito-to-human cycle of transmission. The primary transmitter of the virus is a female mosquito called Aedes aegypti, found in hot regions throughout the world [6,7]. The onset of dengue symptoms is noticed after the incubation (approx. 4–10 days) following the bite of an infected vector [7].

WHO has categorized dengue as Dengue (with or without warning signs) and Severe Dengue [3]. A high fever (104 °F) characterizes the febrile phase of dengue along with two or more of the following symptoms: headaches, pain behind the eyeballs, nausea/vomiting, swollen glands, pain in muscles/bones/joints, and rashes [3,7,8]. The above symptoms are usually seen for 2–7 days following incubation. The critical phase of dengue appears 3–7 days after the initiation of the symptoms. The warning signs include severe abdominal tenderness, continuous vomiting, rapid breathing, mucosal bleeding, lethargy, restlessness, liver enlargement, and blood in vomit/stools [3,7]. Plasma leaking, fluid accumulation, respiratory distress, severe bleeding, or organ failure are the deadly consequences of severe dengue [3,7,8]. Mild dengue is rarely associated with fatal complications. However, severe dengue may lead to death without proper medical care. Hence, early and accurate laboratory investigations and seeking appropriate medical care are crucial for the recovery of dengue patients [3].

Several methods are used for the diagnosis of DENV infection, including the complete blood count (CBC) test, virus isolation methods, serological methods, and peripheral blood smear (PBS) analysis [3,6,7]. Infection in the body brings about a change in the number of leukocytes and platelets. Usually, they are highly lowered in number [9]. Virus isolation is a traditional method used for the diagnosis of DENV infection. However, it has recently been replaced by molecular methods, such as RT-PCR and NS1 antigen-capture ELISAs [7]. RT-PCR methods are considered the gold standard and provide an accurate diagnosis. Nevertheless, it requires specialized laboratory equipment, which is not affordable in remote areas/a low resource setup where dengue is endemic [3,7]. Dengue can also be diagnosed by detecting the presence of viral antigens (NS1) in the blood of dengue-infected patients. This simple but effective diagnostic tool provides qualitative positive/negative results [3,7]. Serological methods, such as IgM and IgG antibody-capture ELISAs, are useful for detecting recent or past DENV infection. IgM and IgG in the blood serum indicate recent and past DENV infections, respectively [3,7]. 

The microscopic examination of PBS images is regarded as the gold standard for investigating various diseases [10]. However, it is labor-intensive, time-consuming, and needs an expert pathologist to interpret the blood smears. Automated image analysis of PBS can expedite diagnosis, reduce inter and intra-observer error, and reduce the pathologists’ load [11,12,13]. This idea falls under the domain of digital pathology. Digital pathology is an image-based environment that allows pathology information management from digital glass slides [14,15]. The high-resolution digital images of slides are viewed and analyzed using automatic image-analysis algorithms, which give precise and reproducible results. Additionally, digital pathology coupled with AI principles has led to the development of novel and innovative diagnostic tools that hold immense potential in improving healthcare services [14,15]. In recent years, various machine/deep learning approaches with advanced medical image processing techniques have been explored for disease diagnosis from PBS [16,17,18,19]. Many researchers have published studies related to the automatic diagnosis of various hematological disorders including leukemia, anemia, and malaria [20,21,22].

Platelet count in patients with dengue fever drops below the normal range (<1,00,000/µL of blood) and can reach as low as <40,000/µL of blood, a condition called thrombocytopenia [9,23]. Thrombocytopenia is common in both mild and severe dengue cases [24,25]. Studies suggest that thrombocytopenia is a significant cause of bleeding in dengue patients [23,26]. Thus, counting platelets is essential to provide early treatment to dengue patients. Studies have shown that the platelet count estimation using PBS is not significantly different from using an automated hematology analyzer (based on the flow cytometry principle) [27,28]. Leukocytes are the immunity cells that help the body to resist infections and other diseases. Each of the five types of leukocytes plays a distinct role. However, lymphocytes are a focus throughout this work. The dengue infection in the body, in turn, alters the lymphocytes’ morphology. Studies show that this alteration is an essential diagnostic clue for dengue diagnosis [29,30,31,32]. Hence, PBS analysis can considerably help the diagnosis of dengue, which can act as a complement to the CBC test and NS1 antigen-capture method [29]. 

**Figure 1:**
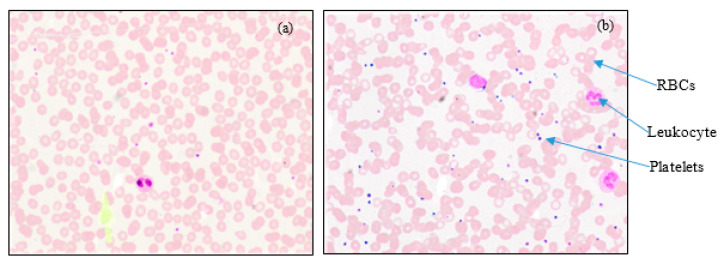
40x digital microscopic PBS images: (**a**) Dengue (few platelets), and (**b**) Normal. (Courtesy: KMC, Manipal).

The typical microscopic images of blood smear with 40x magnification are shown in Figure 1, representing normal and dengue cases. The figure shows fewer platelets in the case of dengue. 

**Figure 2:**
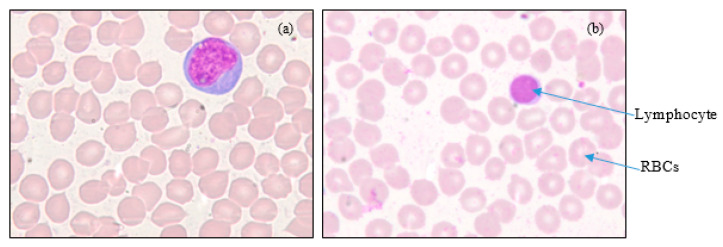
100x digital microscopic PBS images: (**a**) Dengue (reactive lymphocyte), and (**b**) Normal. (Courtesy: KMC, Manipal).

Shown in Figure 2 are 100x images representing normal and dengue cases. 

Morphological changes can be seen in the lymphocytes in the case of dengue. Morphological changes include changes in the nucleus and the cytoplasm of the lymphocytes. The nucleus will become bigger and irregular. Cytoplasm will increase and will become bluer.

Most of the research work reported on automated diagnosis of dengue is by utilizing symptoms, vital signs, blood profile data, or a combination of these [33,34,35,36,37,38,39,40,41]. In this work, features of platelets and lymphocytes from PBS are thought out for the automated detection of dengue fever. Just a few researchers have thought of features of Platelets/lymphocytes for this purpose [42,43,44,45]. However, similar works carried out by various researchers for other studies, including platelet detection and counting and leukocyte segmentation and classification, are discussed below.

Cruz et al. proposed a raspberry-pi-based system to estimate platelet count from microscopic blood smear images. RGB images were converted to HSV color space. After thresholding, morphological operations were performed. Connected component labeling was used to count platelets. They performed statistical analysis to compare this algorithm’s results with the CBC results and reported an accuracy of 90% [46]. Evangeline et al. proposed an algorithm to count platelets from 40x microscopic blood smear images. RGB images were converted into grayscale. Then, the contrast stretched, histogram equalized grayscale images were subjected to Otsu’s thresholding. Edge detection and morphological opening removed platelets from the image and retained only the WBC nuclei. This image was then used as a mask to remove the WBC nuclei and retain only the platelets. The authors reported an accuracy of 91% [47]. Meimban et al. presented a more accurate new algorithm for counting platelets using Python OpenCV. Platelets were counted from 100x blood smear images. RGB images were converted into HSV color space, and platelets were segmented using Otsu’s thresholding. Then the blob detection algorithm was applied to the segmented images, and the platelets were counted. The authors reported an accuracy of 100% [48]. Mahanta et al. developed an Image processing technique to detect and count platelets from blood smear images. RGB images were converted into LAB color space. Morphological operations of opening and dilution were performed after segmentation. Then WBCs were eliminated, and the platelets were counted. The authors reported an accuracy of 95% [49]. Monteiro et al. worked on an image processing algorithm to detect and count platelets from blood smear images. Images were pre-processed, and color converted. Then the Hough transform was applied, and the platelets were counted with an accuracy of 90% [50]. Alam et al. presented an ML approach for automatic identification and counting platelets using the YOLO algorithm. The authors obtained an accuracy of 96% [51]. Although these approaches have yielded good results, most researchers have implemented the algorithms on only a few samples. Moreover, they have not averaged the platelet counts from 10 consecutive fields, which is mandatory. Furthermore, the platelet obtained are not compared with the corresponding count from the hematology analyzer, which is a gold standard.

Manik et al. enhanced the classification of WBCs in PBS images with a new framework. They segmented the nucleus and cells, and extracted morphological/textural features. The NN Pattern Recognition tool was employed to classify the WBCs. Authors reported an accuracy of 98.9% [52]. Sajjad et al. developed a scheme to classify the WBCs in PBS images. They employed K-means clustering to segment the nucleus. Subsequently, they used DWT to extract geometrical/statistical/textural features. An Ensemble-SVM classifier was employed to classify the WBCs. They achieved an accuracy of 94.7% [53]. Shahin et al. proposed a novel CNN architecture named WBCsNet to identify different WBCs. The architecture consisted of three main convolutional layers, two pooling layers, four ReLU units, and two fully-connected layers. An accuracy of 96.1% was obtained, which was better than different transfer learning approaches and traditional identification systems [54]. Hegde et al. presented a technique to find WBCs in PBS. A robust active contour method detected and extracted WBCs using Zack’s thresholded nuclei with an overall sensitivity of 96%. Furthermore, they correlated the conventional and convolution neural network (CNN) concept of WBC classification. They classified the WBCs into normal and abnormal types. In the conventional method, they extracted features, viz., shape, color and texture and classified the WBCs using neural networks. They achieved 99.8% and 99% accuracies for conventional and CNN techniques [55,56]. Banik et al. developed a method to automatically segment the leukocyte nuclei from the blood smear images. Nucleus segmentation was based on HSI & L*a*b color space and K-means clustering, making the method independent of the database. Leukocytes were located based on the location of the segmented nuclei. Then, the cropped leukocytes were classified using CNN. They achieved an average accuracy of 98.61% and 96% for nucleus segmentation and classification [57]. Aziz et al. worked on a CNN-based algorithm for classifying leukocytes in PBS. They used K-means clustering to segment the leukocytes in L*a*b space. They employed pre-trained models—AlexNet and ResNet18 for classification and reported an accuracy of 93.30% and 93.85% for AlexNet and ResNet18, respectively [58]. Sapna et al. reported a concept to classify leukocytes with MLP and SVM. The authors segmented the nucleus by employing Fuzzy C-means clustering. Subsequently, they derived geometrical/color/texture features. They achieved 92.8% and 91.5% accuracy for MLP and SVM, respectively [59]. Togacar et al. used deep features to classify WBCs. They employed feature extractors viz. AlexNet, GoogleNet, and ResNet-50. MIC/Ridge extracted the most relevant features. The WBCs were classified using quadratic discriminant, which earned an accuracy of 97.95% [60]. Cinar et al. developed a hybrid CNN model to classify WBCs. They considered pre-trained—Alexnet and GoogleNet deep features. Subsequently, SVM classified the WBCs. They obtained 99.73% and 98.23% accuracy for databases viz. Kaggle and LISC [61].

This work considers PBS-based features of platelets and lymphocytes for automated dengue fever detection. Just a few articles similar to this work are found in the literature. The dataset consists of 100x digital microscopic PBS images of dengue and normal controls acquired using an Olympus DP25 digital microscope available in the Hematology laboratory, KMC, Manipal. The significant contributions of this paper are: (i) A clinically acceptable blob detection algorithm for the detection of thrombocytopenia in dengue cases; and (ii) Automated dengue detection based on the morphological and GLSDM features extracted from the lymphocyte. In addition, a comparative study of the results with the results obtained by the classifiers considering the deep and LBP features is presented.

The remaining sections of the manuscript are arranged in the following manner: Section 2 illustrates the materials and methods of the proposed system. Then, the results are furnished in Section 3, followed by a brief discussion in Section 4 and the conclusion in Section 5.

## 2. Methodology

**Figure 3:**
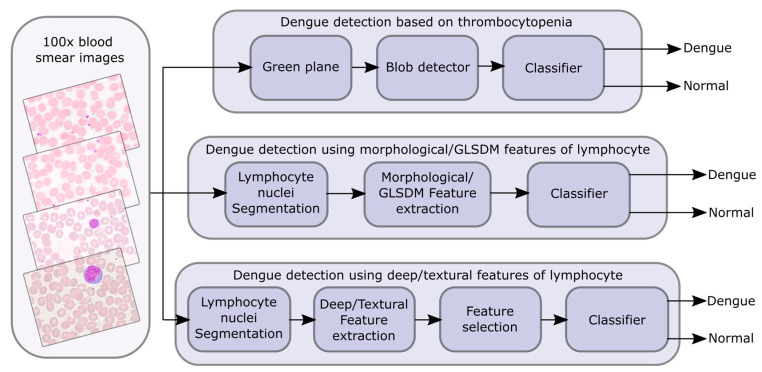
Conceptual layout of the planned dengue detection scheme.

This section is subdivided into four parts. The first part deals with data acquisition. The blob detection algorithm for diagnosing thrombocytopenia is discussed in the next part. Then, in the third part, the detection of dengue by utilizing morphological features/GLSDM-based textural features from the lymphocyte is described. Finally, the last part describes dengue detection using lymphocyte-based MobileNetV2-based deep features/LBP-based textural features. Figure 3. shows the conceptual layout of the planned dengue detection scheme.

### 2.1. Data Acquisition

The KMC and KH Institutional Committee issued ethical clearance (IEC Project No: 114/2020) to acquire the necessary dataset from the Hematology Laboratory, Kasturba Hospital, Manipal. The dataset contains PBS images acquired from 94 blood smear slides of different subjects (54 dengue-infected subjects and 40 normal controls). Hospital numbers of Dengue patients (based on ICD codes) were obtained from the Medical Records Dept., KH, Manipal. The blood smear slide numbers corresponding to the hospital numbers were obtained from the Lab Report Viewer software. A digital microscope (Olympus DP25) extracted the PBS images from the Leishman stained glass slides with a magnification of 100x and resolution of 2560 × 1920. The 100x image is an ‘Oil Immersion Field’ as a drop of Liquid paraffin oil is spread over the slide before the image is captured. This gives us clean images where the ROIs can be clearly identified. The images were captured by focusing sharply on the area between the blood smear slide’s body and tail. The RBCs are scarce and spaced out better in this region than in the body, where many RBCs exist. This makes it easier to identify the platelets and lymphocytes. Segmentation also becomes easier as there is minimal overlapping of the cells in this region.

### 2.2. Diagnosis of Thrombocytopenia Using a Blob Detection Algorithm

A blob detection algorithm was developed using Python OpenCV to detect and count platelets from 100x digital microscopic PBS images. Each component of the RGB image was analyzed individually. The green component was selected for further analysis as the ROIs (platelets) are clearly visible in the image. Then, the blob detector was applied to the green component to detect and count the platelets. Platelets are detected and counted from 10 consecutive oil immersion fields and then averaged.

Python OpenCV provides a convenient way to detect and filter the blobs in an image [48]. A Blob (Binary large object) is a collection of connected pixels that share some common property [62]. In this context, platelets are blobs. The blob detector is controlled by several parameters, including Thresholds and Filters [63]. Filters include Color, Area, Convexity, Circularity, and Inertia [63]. Depending on the application, the default values of the parameters are fine-tuned to obtain desired results [64].

**Figure 4:**
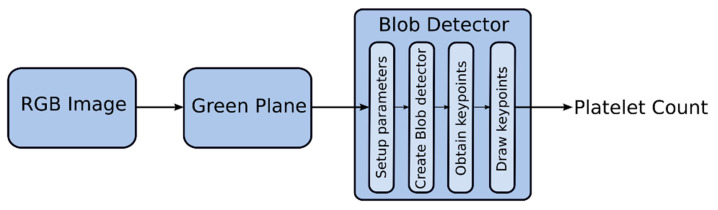
Sequence in the Blob detection algorithm.

Basically, the blob detector converts the image into several binary images by applying thresholds from minThreshold to maxThreshold with a threshold step. First, the center of each blob is calculated, and blobs from several binary images are combined into one group based on the minDistanceBetweenBlobs parameter. Then, the required filters are enabled and applied after fine-tuning its parameters. Returned keypoints contain information regarding the center and diameter of each blob detected. Draw keypoints—draw a circle around the detected blobs. The number of keypoints is equal to the number of blobs [48,62,64]. In this application, maxThreshold was fine-tuned. Filters—Area, Convexity, and Inertia were enabled, and its parameters (minArea, minConvexity, and minInertiaRatio) were fine-tuned. Figure 4 shows the sequence in the Blob detection algorithm.

### 2.3. Detection of Dengue by Utilizing Morphological Features and GLSDM-Based Textural Features from the Lymphocyte Nucleus

This sub-section presents details of the segmentation process of the lymphocyte nuclei, the morphological/GLSDM features extracted, and the different classifiers used to classify normal and dengue-infected smears. PBS images acquired from 94 different subjects, i.e., 54 dengue-infected and 40 normal controls, are included in the study.

#### 2.3.1. Lymphocyte Nuclei Segmentation

The objective here is to extract the lymphocyte nucleus from the background. Various methods are available for segmenting blood smear images, including thresholding, clustering, edge-based, and transform-based [12]. However, threshold-based segmentation methods are used by the majority of researchers [12]. Moreover, K-means and Otsu’s methods are used extensively to segment WBC nuclei [65].

**Figure 5:**
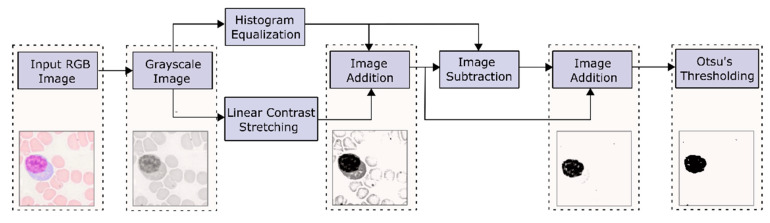
Segmentation process of lymphocyte nucleus.

The flowchart shown in Figure 5 depicts the steps involved in segmenting the lymphocyte nuclei. In this work, Otsu’s global thresholding was utilized for segmentation. Herein, the contrast of the RGB converted greyscale image is enhanced using two techniques, viz. linear contrast stretching and histogram equalization. Subsequently, the contrast-stretched and histogram-equalized images are subjected to necessary arithmetic operations, resulting in a darker nucleus. Then, this image is binarized using Otsu’s thresholding [66].

The segmentation process retains only the area of interest, and all other image components are made part of the background. This made it easier to study the lymphocyte nuclei morphology and to identify six appropriate features for classification.

#### 2.3.2. Feature Extraction

Six distinctive handcrafted morphological features that distinguished normal lymphocytes from the Dengue-infected ones were identified following the segmentation process. The features identified and extracted include Area, Perimeter, Major Axis Length, Minor Axis Length, Eccentricity, and Circularity.

- *Area*

It Indicates the actual number of pixels in the region [67,68].

- *Perimeter*

It Indicates the distance covered along the boundary of the region [67,68].

- *Major Axis Length*

It Indicates the length (in pixels) of the ellipse’s major axis [67].

- *Minor Axis Length*

It Indicates the length (in pixels) of the ellipse’s minor axis [67].

- *Eccentricity*

It measures the ovalness of an ellipse and is given by the ratio of the distance between the ellipse’s foci and its major axis length. The value lies in the range of 0 and 1. An eccentricity of 0 represents a circle, while an eccentricity of 1 represents a line segment [67].

- *Circularity*

It measures the roundness of the object and is given by Equation (1). If the circularity is one, it indicates a perfect circle, and zero indicates a line [67,68].

(1) $$Circularity=\frac{4*\pi *Area}{Perimeter^{2}}$$

In addition, textural features from the GLSDM were also considered. GLSDM is a statistical method that identifies image texture by examining the spatial relationship of pixels in an image [69,70]. GLSDM captures relationships between a pair of pixels by calculating how often a pixel with the gray-level i occurs in a specific spatial relationship to a pixel with gray-level j [70,71]. By default, the spatial relationship is defined as the pixel of interest and the pixel horizontally adjacent. However, other spatial relationships between the two pixels can be considered. The gray levels in the image determine the size of the GLSDM. For an image I of size M×N, the GLSDM is defined by [72,73] Equation (2).

(2) $$C_{d}\left(i,j\right)=\left|\left\{\left(p,q\right),\left(p+\Delta x,q+\Delta y\right):I\left(p,q\right)=i,I\left(p+\Delta x,q+\Delta y\right)=j\right\}\right|$$

Herein,$\;\left(p,q\right),\left(p+\Delta x,q+\Delta y\right)\in M\times N$, $d=\left(\Delta x,\Delta y\right)$ and $\left|.\right|$ represent the cardinality of the set [74,75,76]. The probability of a pixel with gray-level  i having a pixel with gray-level  j at a distance $\left(\Delta x,\Delta y\right)$ away is denoted by Equation (3) [74,75,76].

(3) $$P_{d}\left(i,j\right)=\frac{C_{d}\left(i,j\right)}{\sum_{i,j}C_{d}\left(i,j\right)}$$

The four statistical properties of the image derived from GLSDM were—Contrast, Energy, Correlation, and Homogeneity.

- *Contrast*

It measures intensity variation between a pixel and its neighbors over the entire image. Contrast is 0 if the image is constant. It is computed using Equation (4) [74,77].

(4) $$Contrast=\underset{i,j}{\sum }{\left|i-j\right|}^{2}P_{d}\left(i,j\right)$$

- *Energy*

It is the measure of uniformity and is given by the summation of squared values in the GLSDM. For a constant image, energy is 1. It is computed using Equation (5) [74,77].

(5) $$Energy=\underset{i,j}{\sum }P_{d}{\left(i,j\right)}^{2}$$

- *Correlation*

It measures the similarity of the image texture across the pixels [77]. A perfect positively correlated image has a correlation of 1, and a perfect negatively correlated image has a correlation of −1. It is computed using Equation (6).

(6) $$Correlation=\underset{i,j}{\sum }\frac{\left(i-\mu_{i}\right)\left(j-\mu_{j}\right)P_{d}\left(i,j\right)}{\sigma_{i}\sigma_{j}}$$

Herein, $\mu_{i}$ and $\mu_{j}$ indicate mean along the row and column, respectively. $\sigma_{i}$ and $\sigma_{j}$ indicate standard deviation along the row and column, respectively [77].

- *Homogeneity*

It measures how close the element distribution in the GLSDM is to its diagonal. For a diagonal GLSDM, homogeneity is 1. It is computed using Equation (7) [74,77].

(7) $$Homogeneity=\underset{i,j}{\sum }\frac{P_{d}\left(i,j\right)}{1+\left|i-j\right|}$$

#### 2.3.3. Classification

This work uses six popular supervised classifiers to classify dengue-infected and normal smears. Fine Decision Tree (DT), Linear Discriminant Analysis (LDA), Gaussian Naïve Bayes (NB), Quadratic Support Vector Machine (SVM), Fine K-Nearest Neighbor (KNN), and Narrow Multilayer Perceptron (MLP) was used to differentiate dengue-infected and normal smears. K-fold cross-validation, with K = 10, is used to build and assess the predictive potential of the classifiers. The cross-validation strategy alleviates the overfitting problem [78,79]. A concise explanation of the various classifiers is presented below.

- *Decision Tree (DT)*

DT classifier is a binary tree that recursively splits the data set until it results in pure leaf nodes [80]. Decision nodes contain a condition to split the data, and the leaf nodes predict the class of a new data point. Different methods are used to decide the optimal split [81]. Here, Gini Index, a computationally efficient method, was used.

- *Linear Discriminant Analysis (LDA)*

The LDA classifier maximizes the separability between the classes by projecting the data from higher dimensional feature space to a lower one [82]. The objective here is to simultaneously maximize the distance between the means of the classes and minimize the variance within each class. This objective is met optimally by maximizing Fisher’s Discriminant Ratio (FDR) [82].

- *Naïve Bayes (NB)*

NB is a Bayes theorem-based probabilistic classifier. It is built on the assumption that the features are conditionally independent [83,84].

- *Support Vector Machine (SVM)*

SVM is very powerful and versatile. It finds a hyperplane that greatly segregates the two classes by maximizing the margin between the support vectors [74,83,85]. Linearly inseparable data are handled by SVM classifiers using the kernel trick [74,83,85]. Quadratic SVM, which uses a second-order polynomial kernel, is used in this study.

- *K-Nearest Neighbor (KNN)*

KNN is a non-parametric distance-based classifier [81,86]. A user-defined value K is set, and nearest K neighbors are found based on their distance from the test instance. The most common class among the nearest K neighbors is assigned as the class for the test instance [87].

In this work, K is set to 1, and the Euclidean distance metric was used to determine the neighborhood.

- *Multilayer Perceptron (MLP)*

MLP is a feedforward neural network classifier [88,89]. In this work, ReLU is used as the non-linear activation function. The activation at the output is always Softmax, which produces the predicted classification scores and class labels. The weights of MLP are modified using the backpropagation learning rule based on the Gradient Descent Procedure (GDP) [88]. 

The summary of the parameters used to build the six classifiers is recorded in Table 1.

### 2.4. Detection of Dengue by Making Use of MobileNetV2 Deep Features and LBP Textural Features from the Lymphocyte Nucleus 

The lymphocyte nuclei segmentation was accomplished using Otsu’s global thresholding, as mentioned in Section 2.3.1. Thereafter, deep and handcrafted features were extracted out of the segmented nuclei to distinguish normal and dengue-infected lymphocytes [10]. The deep features were extracted from the fully-connected layer ‘Logits’ of the lightweight pre-trained deep network MobileNetV2. The handcrafted textural features were extracted using the local binary pattern (LBP) technique. Further, the features were ranked and selected using the ReliefF feature selection algorithm [10]. The highly ranked features were fed to various supervised classifiers mentioned in Section 2.3.3. A cross-validation strategy with 10-fold was employed to build and evaluate the predictive potential of the classifiers.

## 3. Results

This section presents the results of thrombocytopenia detection using the blob detection algorithm. It also presents the performance of the classifiers in the automatic detection of dengue from the lymphocytes. In addition, it presents the comparison of classification results based on morphological/GLSDM textural features and deep/LBP textural features.

### 3.1. Results of the Blob Detection Algorithm

**Figure 6:**
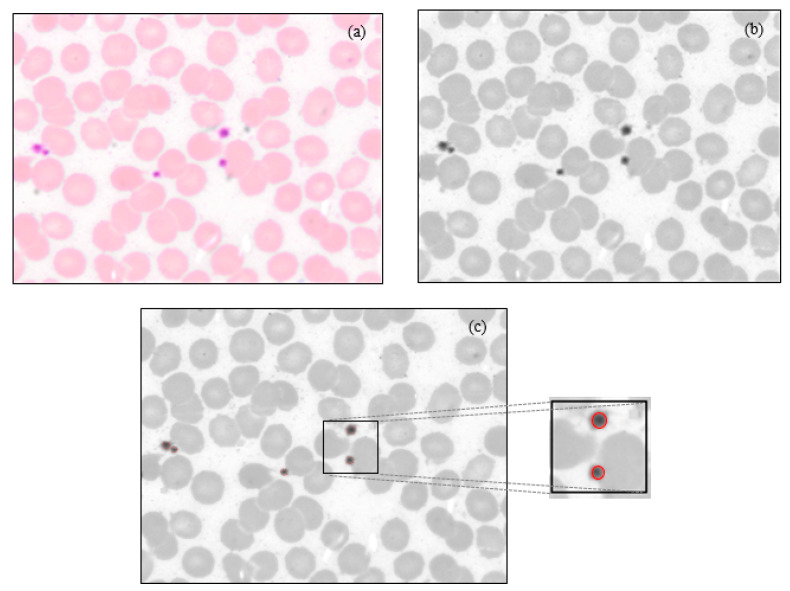
The sample results of the intermediate steps in the platelet detection process: (**a**) Original 100x RGB image, (**b**) Green component, and (**c**) Platelet detection.

Platelets are detected and counted from 10 consecutive oil immersion fields and then averaged. In order to obtain the platelet count per microliter of blood, the average value is multiplied by a factor of 15,000. The calibration factor is used to extrapolate the averaged value to that of a complete microliter of blood. The intermediate results of the algorithm developed to detect platelets from 100x digital microscopic PBS images are shown in Figure 6. 

Table 2 shows the platelet counts per microliter of blood obtained for ten patients and the corresponding machine count. The machine count indicates the ADVIA hematology analyzer’s count. The proposed algorithm achieved an average accuracy of 90%. The results obtained are promising and clinically acceptable. Statistical analysis performed indicates no significant difference between the machine and automated platelet count at a 5% significance level.

### 3.2. Results of Segmentation, Feature Extraction, and Classification for Dengue Detection from the Lymphocyte Nucleus Using Morphological Features/GLSDM-Based Textural Features

**Figure 7:**
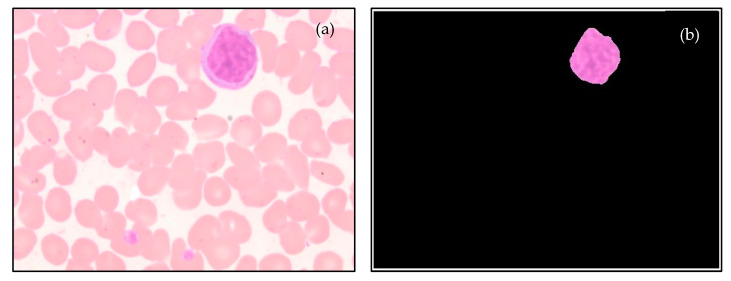
Sample results of segmentation process: (**a**) Dengue-infected lymphocyte, and (**b**) Segmented nucleus.

**Figure 8:**
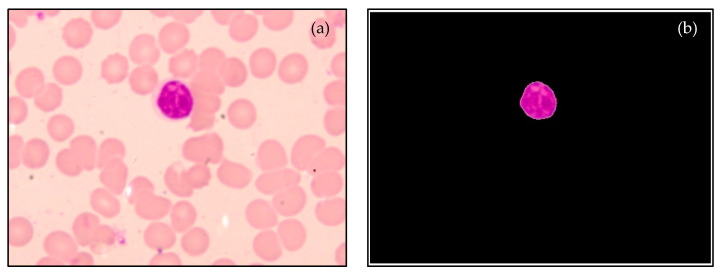
Sample results of segmentation process: (**a**) Normal lymphocyte, and (**b**) Segmented nucleus.

The process of segmenting the lymphocyte nuclei is depicted in Figure 5. Figure 7 and Figure 8 display sample segmentation results for dengue and normal blood smears. The algorithms were implemented using MATLAB (R2021a). 

**Figure 9:**
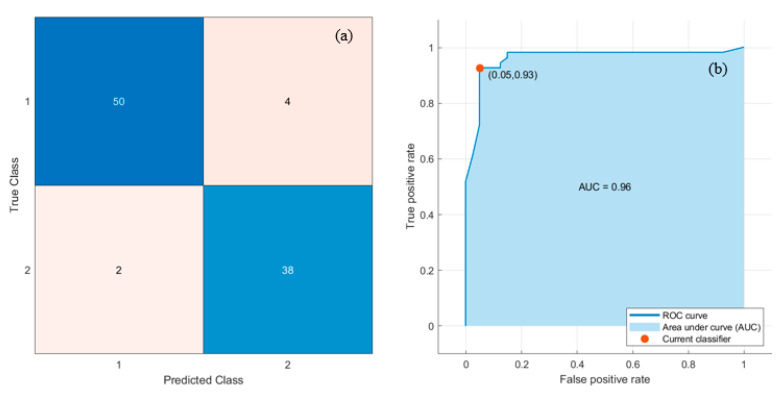
Best results with SVM/DT classifiers: (**a**) Confusion matrix, and (**b**) ROC curve.

After segmentation, six morphological and four GLSDM features, including Area, Perimeter, Major Axis Length, Minor Axis Length, Eccentricity, Circularity, Contrast, Energy, Correlation, and Homogeneity were extracted out of the nucleus of normal and dengue-infected lymphocytes. Then, a feature matrix was created using Microsoft Excel with columns as the different features and rows as the different samples. Subsequently, the feature matrix was normalized and fed to the classifiers. Six classifiers (DT, LDA, NB, SVM, KNN, and MLP) were trained and tested. The MATLAB Classification Learner Toolbox (MCLT) was employed to implement the classifiers. The best result was obtained using SVM and DT classifiers. The hyper-parameters used to build these six classifiers are recorded in Table 1. Figure 9 depicts the Confusion matrices and Receiver operating characteristic (ROC) curves that were derived from SVM/DT classifiers with 10-fold cross-validation. In the confusion matrix, ‘1’ denotes dengue-infected class, and ‘2’ denotes normal class.

**Figure 10:**
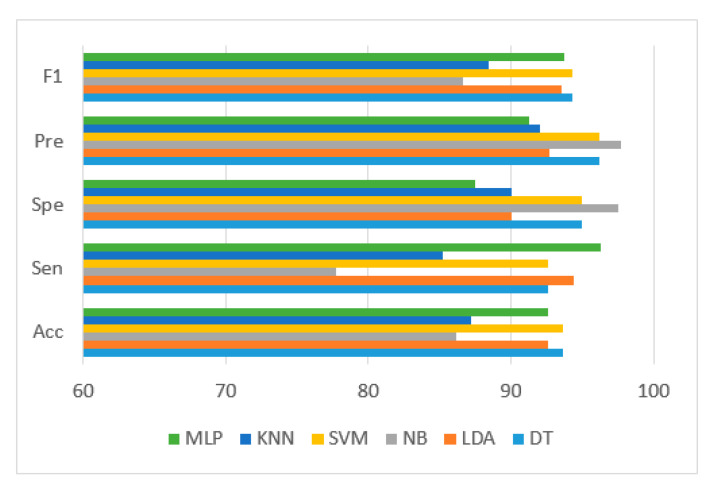
Performance comparison of the classifiers in terms of metrics derived from the confusion matrix.

Six popular performance metrics (Accuracy (Acc), Sensitivity (Sen), Specificity (Spe), Precision (Pre), F1-score (F1), and area under the ROC curve (AUC)) were adopted to assess the performance of the classifiers. Table 3 lists these performance measures and the corresponding AUC values achieved by the six classifiers. SVM/DT classifiers yielded the best results with Acc, Sen, Spe, Pre, F1, and AUC of 93.62%, 92.59%, 95%, 96.15%, 94.34%, and 0.96, respectively, as indicated in Table 3. Moreover, MLP/LDA classifiers achieved second-best results with an Acc of 92.55%, as shown in Table 3. Figure 10 depicts the comparison of the performance of the classifiers based on the metrics derived from the confusion matrices.

### 3.3. Results of Feature Extraction and Classification for Dengue Detection from the Lymphocyte Nucleus Employing MobileNetV2-Based Deep Features and LBP-Based Textural Features

**Figure 11:**
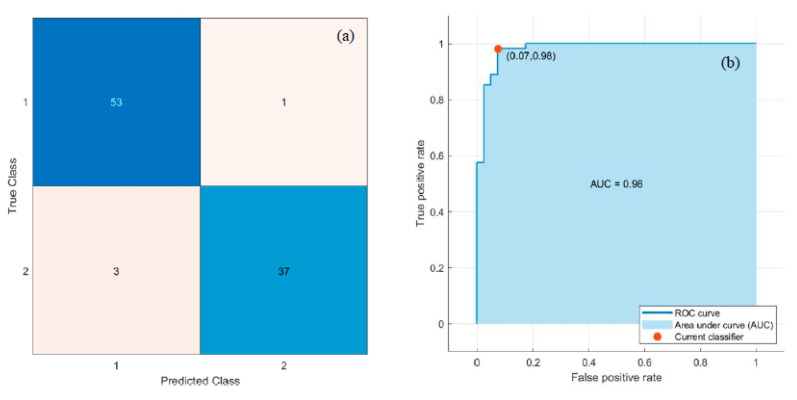
Best results with SVM Classifier: (**a**) Confusion matrix, and (**b**) ROC curve.

**Figure 12:**
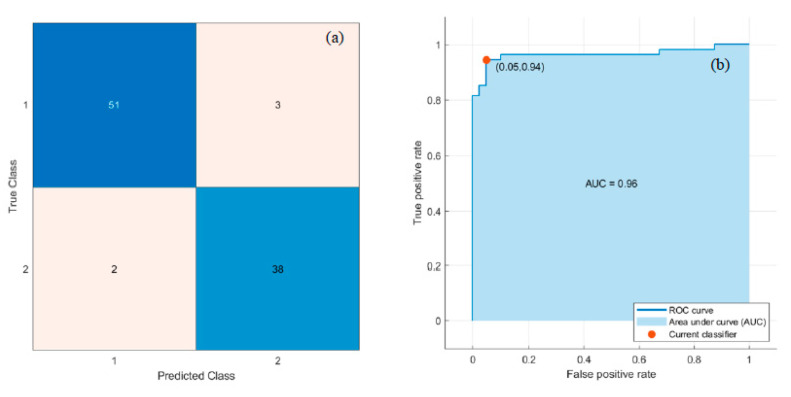
Second best results with MLP classifier: (**a**) Confusion matrix, and (**b**) ROC curve.

Deep and handcrafted features were drawn out from the segmented lymphocyte nuclei. The ‘Logits’ layer of Pre-trained MobileNetV2 produced 1000 deep features, and LBP generated 59 textural features from each component of the RGB image. Thus, a total of 1177 features were generated. The ReliefF feature selection algorithm selected the most discriminative 100 features. Eventually, these 100 features were applied to the six classifiers mentioned in Section 2.3.3 to classify the data into normal and dengue-infected. Classifiers were trained and tested in the same manner as described in Section 3.2. SVM and MLP classifiers, respectively, obtained the best and second-best results. Figure 11 depicts the Confusion matrix and ROC curve obtained for the SVM classifier. In addition, Figure 12 represents the Confusion matrix and ROC curve for the MLP.

Table 4 lists the performance measures achieved by the six classifiers. The best classification performance was achieved using an SVM classifier with Acc, Sen, Spe, Pre, F1, and AUC of 95.74%, 98.15%, 92.50%, 94.64%, 96.36%, and 0.98, respectively, as mentioned in Table 4. Moreover, the second-best results were achieved using MLP, which yielded Acc, Sen, Spe, Pre, F1, and AUC of 94.68%, 94.44%, 95%, 96.23%, 95.33%, and 0.96, respectively, as mentioned in Table 4. 

**Figure 13:**
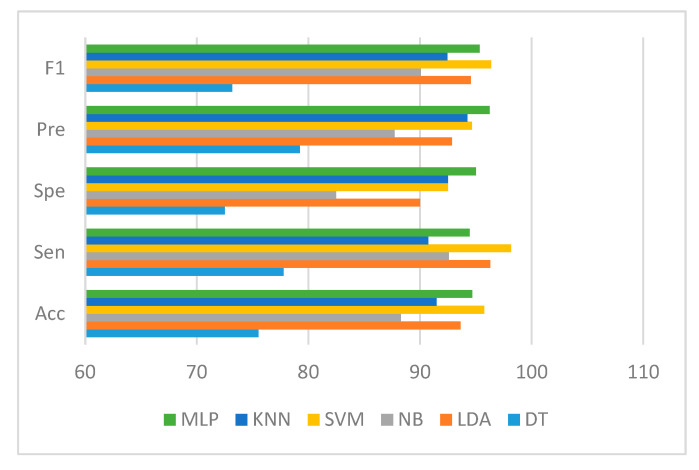
Performance comparison of the classifiers in terms of metrics derived from the confusion matrix.

Figure 13 depicts the comparison of the performance of the classifiers based on the metrics derived from the confusion matrices.

## 4. Discussion

The microscopic examination of PBS remains the gold standard in diagnosing numerous hematological conditions. However, it requires expertise and time. The main goal of this research work is to automate the PBS analysis to assist clinicians in diagnosing dengue. Previous studies related to this topic do not exist in the literature. Hence, this work fills the gap in the literature.

PBS images of dengue are mainly characterized by thrombocytopenia and morphological changes in the lymphocytes. The core component of the research is the classification of dengue and normal controls based on the features extracted from the lymphocyte nucleus. Ten features were extracted from the nucleus, including morphological and GLSDM features. These features, coupled with SVM/DT classifier, achieved the best classification with Acc, Sen, Spe, Pre, F1, and AUC of 93.62%, 92.59%, 95%, 96.15%, 94.34%, and 0.96, respectively. A comparative study of these results was conducted with the results obtained by the classifiers considering the deep and LBP textural features of the nucleus. Deep and LBP features coupled with the SVM classifier yielded the best classification with Acc, Sen, Spe, Pre, F1, and AUC of 95.74%, 98.15%, 92.50%, 94.64%, 96.36%, and 0.98, respectively. While, these features coupled with MLP yielded the second-best classification with Acc, Sen, Spe, Pre, F1, and AUC of 94.68%, 94.44%, 95%, 96.23%, 95.33%, and 0.96, respectively. Deep and LBP features yielded marginally better results. Although a limited dataset is used, this study successfully contributes to the classification of the dataset.

A blob detection algorithm was also developed to diagnose thrombocytopenia in dengue cases using Python OpenCV. The results obtained were statistically significant. Different approaches are available in the literature to detect thrombocytopenia from PBS images, as discussed in Section 1. Unlike other approaches, which obtained platelet counts from only one field, this approach obtained platelet counts from 10 consecutive fields and averaged, which is a mandatory procedure. In addition, the platelet counts are compared with the hematology analyzer, a gold standard.

Most of the research work reported on the automated diagnosis of dengue is by utilizing symptoms, vital signs, blood profile data, or a combination of these [90]. A comparative summary of this work with those studies is presented in Table 5. However, the dataset used varies across the studies.

Gambhir et al. proposed a PSO-optimized ANN for the diagnosis of dengue. With 16 attributes, containing symptoms, vital signs, and blood profile data, they classified the data into dengue-positive and dengue-negative and reported an accuracy of 87.27%. Mello-Roman et al. developed a symptom-based diagnostic model for dengue fever. With 38 attributes, including symptoms, they classified the data using MLP and reported an accuracy of 96%. Katta et al. used symptoms to develop an efficient model for dengue detection. The RF classifier yielded an accuracy of 94.39%. Although these studies provided good performance, most of them have not reported cross-validation. Moreover, our study is an entirely different approach based on PBS digital images. The dataset used by us is unique. We have systematically collected authentic hospital data using an Olympus DP25 digital microscope setup. However, this type of dataset is not available publicly. We have not found a similar study in the literature to the best of our knowledge, except for our previous work (Mayrose et al.), indicated in Table 5. The proposed work in this paper yielded marginally lower performance compared to our previous work. However, we have achieved it with a smaller number of features.

## 5. Conclusions and Future Work

Dengue fever is a threat to humans of all age groups. This research aimed to automate the detection of dengue fever from PBS images using advanced ML techniques. PBS images are considered the gold standard for diagnosing various pathological conditions. Currently, AI is being widely used to accelerate research in the biomedical field. Therefore, the synergy of digital pathology and AI could lead to innovative diagnostic tools that provide a more competent diagnostic strategy for assisting pathologists.

Based on the results obtained, the proposed approach could undoubtedly contribute to the automated detection of dengue fever. This research can complement the CBC test/NS1 antigen-capture method and has promising potential in low-resource setup. However, the dataset contained only 94 subjects, and more subjects should be involved to further validate the efficacy of the proposed methodology. The future scope could involve the derivation of features from the lymphocyte cytoplasm in addition to nucleus features. This work also intends to involve pre-trained CNNs for classification purposes in due course.

## Figures and Tables

**Table 1:** Summary of parameters used to build the classifiers.

Classifier	Parameters	Value
DT	Preset Splits Split Criterion Surrogate Decision Splits	Fine Tree 100 Gini’s Diversity Index Off
LDA	Preset Covariance Structure	Linear Discriminant Full
NB	Preset Distribution of Numeric Predictors	GaussianNaive Bayes Gaussian
SVM	Preset Kernel Function Kernel Scale Box Constraint Level Standardize Data	Quadratic SVM Quadratic Automatic 1 True
KNN	Preset Neighbors Distance Metric Distance Weight Standardize Data	Fine KNN 1 Euclidean Equal True
MLP	Preset Fully Connected Layers First Layer Size Activation Iteration Limit Standardize Data	Narrow Neural Network 1 10 ReLU 1000 Yes

**Table 2:** Platelet counts per microliter of blood.

Patient Number	Machine Count	Algorithm Count
1	42,000	45,000
2	113,000	108,000
3	35,000	36,000
4	41,000	31,500
5	80,000	81,000
6	8000	9000
7	16,000	18,000
8	18,000	13,500
9	73,000	76,500
10	61,000	67,500

**Table 3:** Performance measures achieved by the classifiers trained and tested with morphological/GLSDM features.

Classifier	*Acc* (%)	*Sen* (%)	*Spe* (%)	*Pre* (%)	*F1* (%)	*AUC*
DT	93.62	92.59	95.00	96.15	94.34	0.96
LDA	92.55	94.44	90.00	92.73	93.58	0.96
NB	86.17	77.77	97.50	97.67	86.59	0.97
SVM	93.62	92.59	95.00	96.15	94.34	0.96
KNN	87.23	85.19	90.00	92.00	88.46	0.88
MLP	92.55	96.30	87.50	91.23	93.70	0.94

**Table 4:** Performance measures achieved by the classifiers trained and tested with Deep/LBP features.

Classifier	Acc (%)	Sen (%)	Spe (%)	Pre (%)	F1 (%)	AUC
DT	75.53	77.78	72.50	79.25	73.18	0.80
LDA	93.62	96.30	90.00	92.86	94.55	0.93
NB	88.30	92.59	82.50	87.72	90.09	0.94
SVM	95.74	98.15	92.50	94.64	96.36	0.98
KNN	91.49	90.74	92.50	94.23	92.45	0.92
MLP	94.68	94.44	95.00	96.23	95.33	0.96

**Table 5:** Comparative summary of this study with other existing methods.

Article	Attributes	Method	Validation	Acc (%)
Gambhir et al. (2017) [41]	16 features (symptoms, vital signs, and blood profile data)	PSO-ANN ANN	10-fold cross-validation	87.27 79.09
Mello-Roman et al. (2019) [40]	38 features (symptoms)	MLP SVM	90:10 split test	96.00 92.00
Katta et al. (2021) [91]	Symptoms	RF Adaboost M1	64:36 split test	94.39 92.90
Hoyos et al. (2022) [92]	22 features (symptoms, vital signs, and blood profile data)	FCM	10-fold cross-validation	89.40
Mayrose et al. (2021) [10]	100 Deep/LBP features of Lymphocyte nuclei from PBS	SVM MLP	10-fold cross-validation	95.74 94.68
Proposed work (2022)	10 Morphological/GLSDM features of Lymphocyte nuclei from PBS	SVM DT	10-fold cross-validation	93.62 93.62

## Data Availability

The data that support the findings of this study are available from the corresponding author upon reasonable request. The data is not publicly available due to privacy or ethical restrictions.
